# Longitudinal Characterization of the Gut Bacterial and Fungal Communities in Yaks

**DOI:** 10.3390/jof7070559

**Published:** 2021-07-14

**Authors:** Yaping Wang, Yuhang Fu, Yuanyuan He, Muhammad Fakhar-e-Alam Kulyar, Mudassar Iqbal, Kun Li, Jiaguo Liu

**Affiliations:** 1 Institute of Traditional Chinese Veterinary Medicine, College of Veterinary Medicine, Nanjing Agricultural University, Nanjing 210095, China; wangyp96@sina.com; 2 MOE Joint International Research Laboratory of Animal Health and Food Safety, College of Veterinary Medicine, Nanjing Agricultural University, Nanjing 210095, China; 3 College of Veterinary Medicine, Huazhong Agricultural University, Wuhan 430070, China; fyh19970405@163.com (Y.F.); 13545065525@163.com (Y.H.); fakharealam786@hotmail.com (M.F.-e.-A.K.); mudassar.iqbal@webmail.hzau.edu.cn (M.I.); 4 Faculty of Veterinary and Animal Sciences, The Islamia University of Bahawalpur, Bahawalpur 63100, Pakistan

**Keywords:** bacterial microbiota, fungal communities, natural aging, yaks, high-throughput sequencing

## Abstract

Development phases are important in maturing immune systems, intestinal functions, and metabolism for the construction, structure, and diversity of microbiome in the intestine during the entire life. Characterizing the gut microbiota colonization and succession based on age-dependent effects might be crucial if a microbiota-based therapeutic or disease prevention strategy is adopted. The purpose of this study was to reveal the dynamic distribution of intestinal bacterial and fungal communities across all development stages in yaks. Dynamic changes (a substantial difference) in the structure and composition ratio of the microbial community were observed in yaks that matched the natural aging process from juvenile to natural aging. This study included a significant shift in the abundance and proportion of bacterial phyla (Planctomycetes, Firmicutes, Bacteroidetes, Spirochaetes, Tenericutes, Proteobacteria, and Cyanobacteria) and fungal phyla (Chytridiomycota, Mortierellomycota, Neocallimastigomycota, Ascomycota, and Basidiomycota) across all development stages in yaks. As yaks grew older, variation reduced, and diversity increased as compared to young yaks. In addition, the intestine was colonized by a succession of microbiomes that coalesced into a more mature adult, including *Ruminococcaceae_UCG-005*, *Romboutsia*, *Prevotellaceae_UCG-004*, *Blautia*, *Clostridium_sensu_stricto_1*, *Ruminococcus_1*, *Ruminiclostridium_5*, *Rikenellaceae_RC9_gut_group*, *Alloprevotella*, *Acetitomaculum*, *Lachnospiraceae_NK3A20_group*, *Bacteroides*, *Treponema_2*, *Olsenella*, *Escherichia-Shigella*, *Candidatus_Saccharimonas*, and fungal communities *Mortierella*, *Lomentospora*, *Orpinomyces*, and *Saccharomyces*. In addition, microorganisms that threaten health, such as *Escherichia-Shigella*, *Mortierella*, *Lomentospora* and *Hydrogenoanaerobacterium*, *Corynebacterium_1*, *Trichosporon*, and *Coprinellus*, were enriched in young and old yaks, respectively, although all yaks were healthy. The significant shifts in microflora composition and structure might reflect adaptation of gut microbiome, which is associated with physicochemical conditions changes and substrate availability in the gut across all development periods of yaks.

## 1. Introduction

Among the domesticated ruminants, yak (*Bos grunniens*) entered human life as a unique and special species endemic to the plateau (>3000 m) for over 7000 years. It has adapted to the extreme living conditions at low temperature and hypoxia [1]. This ancient species promotes local human civilization and agriculture by providing basic resources for survival on the plateau, e.g., transportation, warm hides, and dung for fuel [2]. In this context yaks may have evolved a mechanism to adapt to their tough living conditions. The ability to adapt in yaks has been studied using the microbiota of the gastrointestinal tract. The trillions of gut microbiota cells regulate health and act as a bridge between the diet and the physiological performance of the host [3,4]. Furthermore, digestive anatomy and physiology change likely distinguish cattle-like ruminants from other ruminant research models, such as giraffes and bovines, not simply in terms of survival habitat [5,6]. Under the same diet conditions, more predicted functions were found in yaks’ gut microbiota than cattle species, indicating more microbiome pathways exist in yaks to adapt to the harsh environment [7]. In addition, the propionate, butyrate, and acetate to propionate ratios were different in donor animals of cow and sheep [8]. It could be due to various reasons, including the microbial composition of the gastrointestinal tract, species, intestinal histological patterns, adaptations to the natural diet, etc. Recent studies have shown the relationship between gut microbiota and the evolution of the host ecology. The gut microbiome can reveal evolutionary genetic changes at the host and microbial cell levels. Numerous aspects of host physiology, such as health status, stress tolerance, and behavior, are closely related to the gut microbiome [9,10,11].

The structure and composition of the initial intestinal microbes of animals originate in the uterus [12] and is significantly affected by microbial exposures at birth, such as the birth canal [13]. Early colonization of the intestinal microbiota is a dynamically evolving, a non-fixed structure influenced by factors such as diet and growth environment [14,15]. Many factors, including the gut physiological environment, weaning, diet, physical activity, and environmental conditions, can have collateral impacts on gut microbiota development as animals grow older. Contemporary studies of the intestinal microbiome exposed a series of novel findings and open questions. Several key components, the taxa *Faecalibacterium*, *Roseburia*, *Subdoligranulum*, and *Escherichia*, might provide the key to disentangling the systemic microbiome-linked immune diseases [16]. Several microbiome members induced the development of Th17 cells, whereas other microbes contributed to regular the development of T cells and intestinal immune tolerance [17]. For ruminants, digestive properties drove unique microbial groups to adapt to high-fiber content foods [18].

In animal husbandry, juvenile ruminants are susceptible to diarrhea, which is mostly related to gastrointestinal dysfunction. Changes in the structure and composition of the intestinal flora during this period have long-term physiological effects on the host [19]. Several studies have shown that the animal’s gut microbiota fluctuates during early development and reaches stability after maturity [20,21]. The diversity of the gastrointestinal micro-ecology increases with age and helps in the maturation of the composition and structure of gut microbiota [22]. Additionally, it evolves into a more restricted ecological niche [23]. Therefore, there might be some unavoidable associations between changes in age and the tolerance against diseases of ruminants. Still, the specific associations and characteristics are unclear. Besides, some potential connections might be present between the age factor and gut microbiota change. However, a report on the potential law between the age-related factor and the changes in the intestinal micro-ecology of the yak is rare.

Based on fungal-bacterial correlation studies, bacterial and fungal populations share a similar intestinal environment. It indicates the integral ecological interactions across entire development stages [24,25,26]. The significant links between intestinal fungal populations and host health have been revealed in recent years [26,27]. Diarrhea was previously reported in yak associated with dysbiosis of intestinal fungi [28]. The eukaryotic community (the mycobiome) coexists with various bacteria and virus in the gut, substantially expanding the repertoire of organisms. Interactions between this commensal and intestinal immune systems have emerged as key for establishing an intestinal balance condition [29,30]. However, the analysis of fungal communities in the gastrointestinal tract of yak remains poorly understood.

Therefore, we hypothesized that the age might influence intestinal microbiota. Elucidating this link between the host and intestinal microbiota was necessary because of the commonly acknowledged association between gut microbiome from juvenile to natural aging and the risks of various diseases [16]. The dynamic distribution of intestinal bacterial and fungal populations in yaks from juvenile to natural aging was identified in this study, characterized by 16S rDNA (16S ribosomal DNA) and ITS (Internal Transcribed Spacer) high-throughput sequencing methods. This study enhanced knowledge about the developmental regulations of intestinal microorganisms in yaks and insights regarding adaptability, nutrition management, and ruminant feeding strategy.

## 2. Materials and Methods

### 2.1. Animals and Samples Collection

A total of 18 yaks (half male and half female) were obtained from high plateau (>3000 m, Qinghai, China), including six young kids (1 year old, Y group), six adults (5 years old, A group), and six older adults (12 years old, O group). The yaks’ body weight and gender information are listed in Table S1. All selected animals received similar immunization procedures and were free of any illness. All yaks grazed freely in the pasture without any dry matter addition. Fresh feces from each yak were collected and rapidly frozen in liquid nitrogen before storing at −80 °C for later examination. The animal-specific procedures were approved by the ethics committee of Animal Experiment Center, Huazhong Agricultural University Wuhan, China (approval No. 4200696959, approval date: 1 December 2020).

### 2.2. DNA Extraction

The QIAamp DNA Mini Kit (QIAGEN, Hilden, Germany) was used to extract genomic DNA, including bacterial and fungal genomic DNA, by following the manufacturer’s instructions. The quality and integrity of collected DNA were assessed by 0.8% agarose gel electrophoresis, while the concentrations were tested by UV-Vis spectrophotometer (NanoDrop 2000, Thermo Scientific, Waltham, MA 02451, USA).

### 2.3. 16S rDNA and ITS Genes Amplification and High-Throughput Sequencing

The specific primers of the bacterial 16S rDNA gene and fungal ITS gene were used for PCR amplification to construct DNA libraries for further sequencing and amplified bacterial V3/V4 regions with primers (338F: ACTCCTACGGGAGGCAGCA and 806R: GGACTA CHVGGGTWTCTAAT). The melting temperature was 55 °C for the 30 s, and PCR cycles were 30. The ITS gene PCR was conducted using primers 5′-NNNNNNNNGCATCGATGAAGAACGCAGC-3′ and 5′-TCCTCCGCTTATTGATATGC-3′ [31]. The annealing temperature was 58 °C during the 35 PCR cycles. PCR primer barcodes were used to facilitate the output of segregated sequencing data. All the PCR products were evaluated and purified by 1% gel electrophoresis and AMPure XP beads (AGENCOURT) to delete the unspecific products. According to the manufacturer’s instructions, Illumina HiSeq 2500 (Illumina, San Diego, CA, USA) used the purified PCR amplification products to generate a sequencing library. The libraries were then treated for quality inspection by keeping only a single peak with the more than 2 nM concentration. Finally, the qualified libraries were used for high-throughput sequencing.

### 2.4. Bioinformatics and Statistical Analysis

In order to obtain more reliable and high-quality sequencing results (effective reads), the following pre-procedures were performed on the raw reads from the Illumina HiSeq platform: (1) Trimmomatic v0.33 software was used to filter the raw reads, and then we identified and removed primer sequences by using cutadapt 1.9.1 software to obtain high-quality reads; (2) in order to get clean reads, FLASH v1.2.7 software was used to splice high-quality reads of all samples through overlap, (3) Chimera sequences were identified and removed by using UCHIME v4.2 software to obtain the final effective reads. Subsequently, the obtained sequences were clustered into OTUs (operational taxonomic units) at over 97% sequence similarity by Usearch software. We used R software (v3.0.3) to draw Venn diagrams, which could visually display the number of common and unique OTUs between different groups. Five indicators, including Chao1, Ace, Shannon, Simpson, and Coverage, were calculated using QIIME2 software to evaluate alpha diversity; it reflected the sample species richness and species diversity. Rarefaction curve and Shannon index curve were used to detect whether the sequencing depth of the sample covered most of the species’ information. The similarities between individuals or groups were assessed using UPGMA (unweighted pair-group method with arithmetic mean) and PCA (principal component analysis). LEfSe (linear discriminant analysis (LDA) effect size) could find biomarkers with statistical differences between different groups. The Metastats software was used to perform a T-test for detecting the difference in the abundance of microbial communities between groups of samples. The values were presented as the mean ± SD. A level of *p*-value < 0.05 were considered statistically significant. In addition, Silva and Unite databases were used for bacterial and fungal classification, respectively.

## 3. Results

### 3.1. 16S rDNA and ITS Sequence Data Analysis

After sequence quality filtering, we obtained 1,393,035 and 1,375,201 clean reads of the V4 region and ITS region in 36 fecal samples. Each sample of bacterial and fungal populations generated an average of 77,391 and 76,400 clean reads, respectively (Tables S2 and S3).

**Figure 1:**
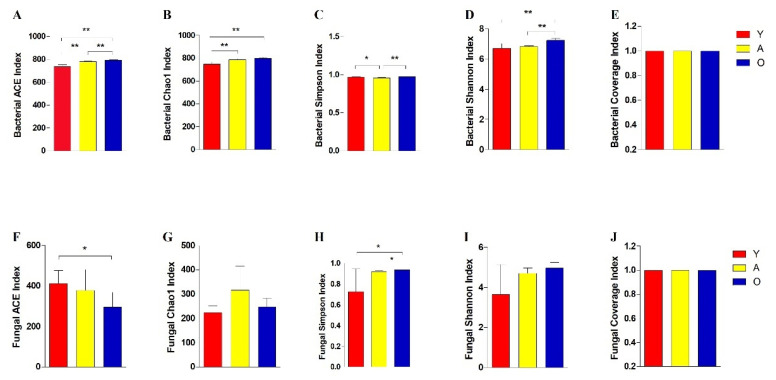
The diversity indices of bacterial and fungal communities in all groups. (**A**–**E**) Represents bacterial ACE, Chao1, Simpson, Shannon, and Coverage indices, respectively. (**F**–**J**) Represents fungal ACE, Chao1, Simpson, Shannon, and Good’s Coverage indices, respectively. * *p* < 0.05; ** *p* < 0.01.

Both multi-samples Rarefaction and Shannon curves tended to be stable, indicating that most bacterial and fungal populations were detected with the current sequencing depth (Figure S1A–D). All estimated Coverage values were over 99%, which indicated the current sequences sufficiently covered the diversity of the sample of bacterial and fungal communities (Figure 1).

**Figure 2:**
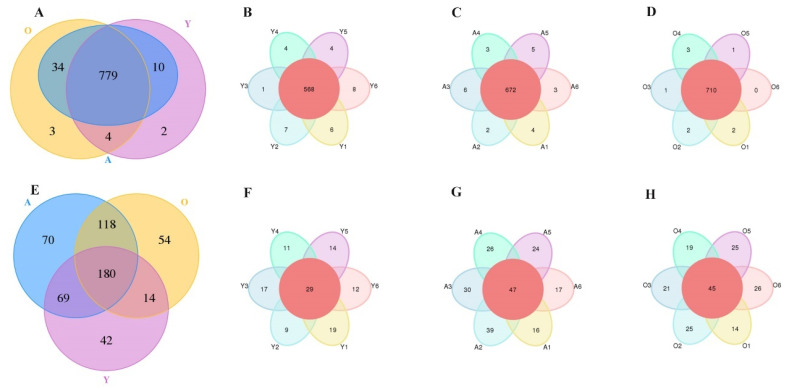
Venn diagram is showing OTUs compositions. (**A**) OTUs of bacterial compositions in all groups; (**B**–**D**) represents the compositions of bacterial OTUs within Y, A, and O groups, respectively. (**E**) OTUs of fungal compositions in all groups; (**F**–**H**) represents the compositions of fungal OTUs within Y, A, and O groups, respectively.

A total of 13,479 and 2995 operational taxonomic units (OTUs), clustered at 97% sequence similarity, were detected in bacterial and fungal feces samples, respectively (Figure 2A,E). Among them, 779 bacterial and 180 fungal core OTUs were recognized in all samples. Moreover, flower Venn showed the numbers of common OTUs were far more than the unique OTUs of each group, suggesting that the sequenced differences of samples between groups were more significant than those within groups. The samples collected had sufficient uniformity (Figure 2B–D,F–H).

### 3.2. Analysis of Bacterial and Fungal Microbiome Diversity with Age

Multiple alpha diversity indices were used to assess the bacterial and fungal populations’ richness and diversity, as measured by ACE, Chao1, Shannon, and Simpson, and they varied significantly with age (Figure 1). The bacterial results showed that ACE and Chao1 indices, which reflect microbiome communities’ richness, significantly increased with age (*p* < 0.01). The richness study of fungal microbiome communities indicated a pattern that differed from that of bacterial microbiome communities. Richness and evenness were also reflected in Simpson and Shannon’s indexes. The elder group also exhibited wider variety in fungal communities.

**Figure 3:**
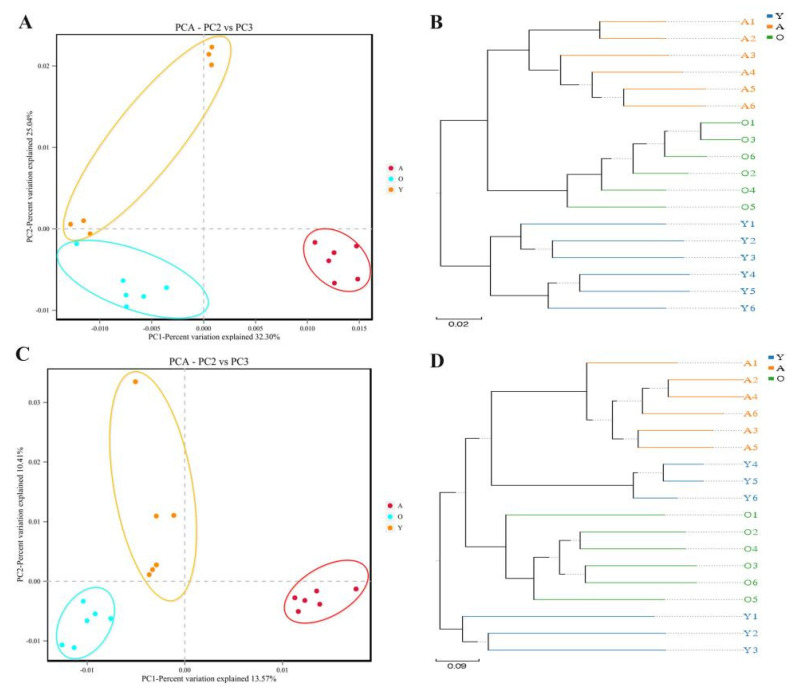
Analysis of bacterial and fungal communities’ structures in all groups. (**A**,**B**) Represents the bacterial similarity between individuals or groups by using PCA and UPGMA, respectively. (**C**,**D**) Represents the fungal similarity between individuals or groups by using PCA and UPGMA, respectively.

To assess beta diversity, we used UPGMA, which reflect variations in samples in microbial communities in the evolutionary tree and PCA. Three branches were segregated with age based on variations in evolutionary information of each sample (Figure 3B,D). As shown in the scatterplot from PCA, the three developmental stages of yaks showed continuous alterations in bacterial communities. Moreover, scattered points in the Y group of bacterial communities were more scattered, suggesting variability and uncertainty in the bacterial microbiome of the yak calves (Figure 3A). Fungal communities were sequestered into three clusters according to age, indicating a difference in gut fungal communities between old, adult, and calf yaks (Figure 3C).

### 3.3. Significant Alterations in Gut Bacterial Microbiota Composition with Age

**Figure 4:**
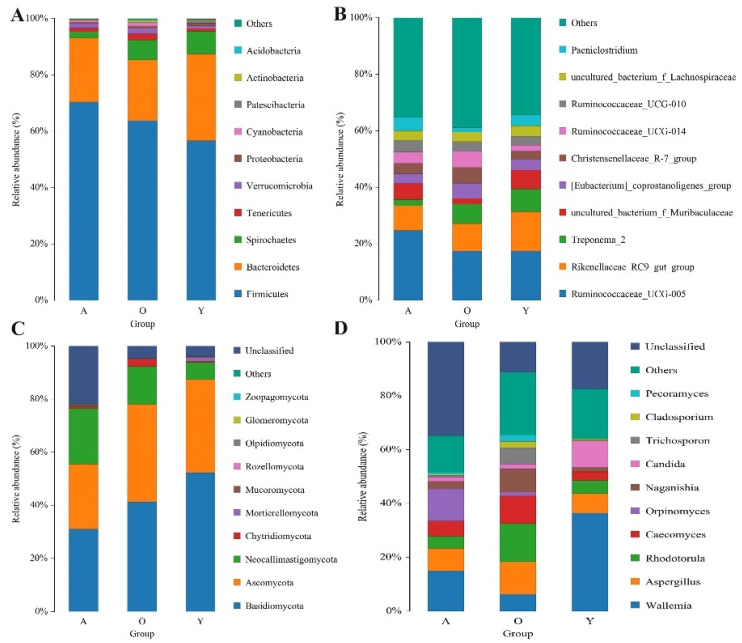
Histogram of the distribution of the top 10 species in abundance. (**A**,**B**) Showed bacterial abundance at the taxonomic level in phyla and genera, respectively; (**C**,**D**) showed fungal abundance at the taxonomic level in phyla and genera.

**Figure 5:**
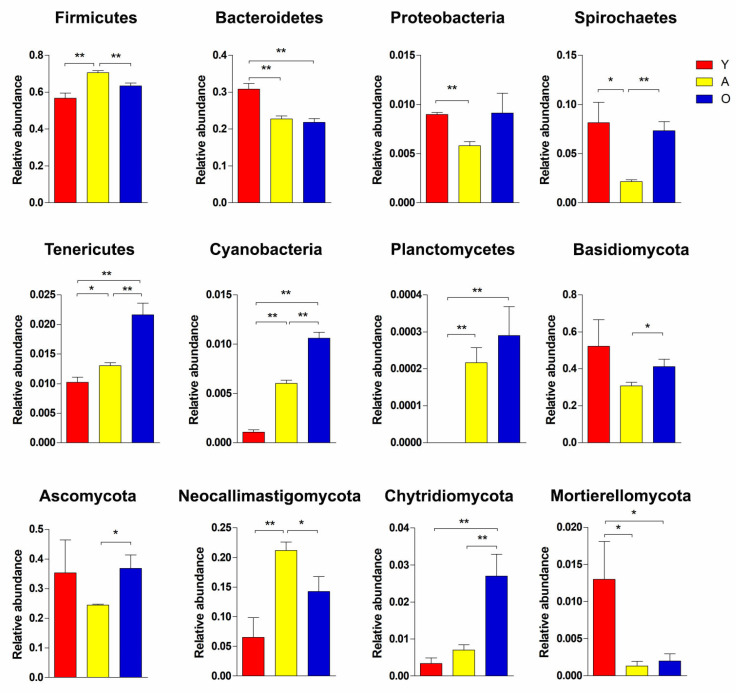
The differences in the relative abundance of bacteria and fungi between three development stages at the phylum level. * *p* < 0.05; ** *p* < 0.01.

**Figure 6:**
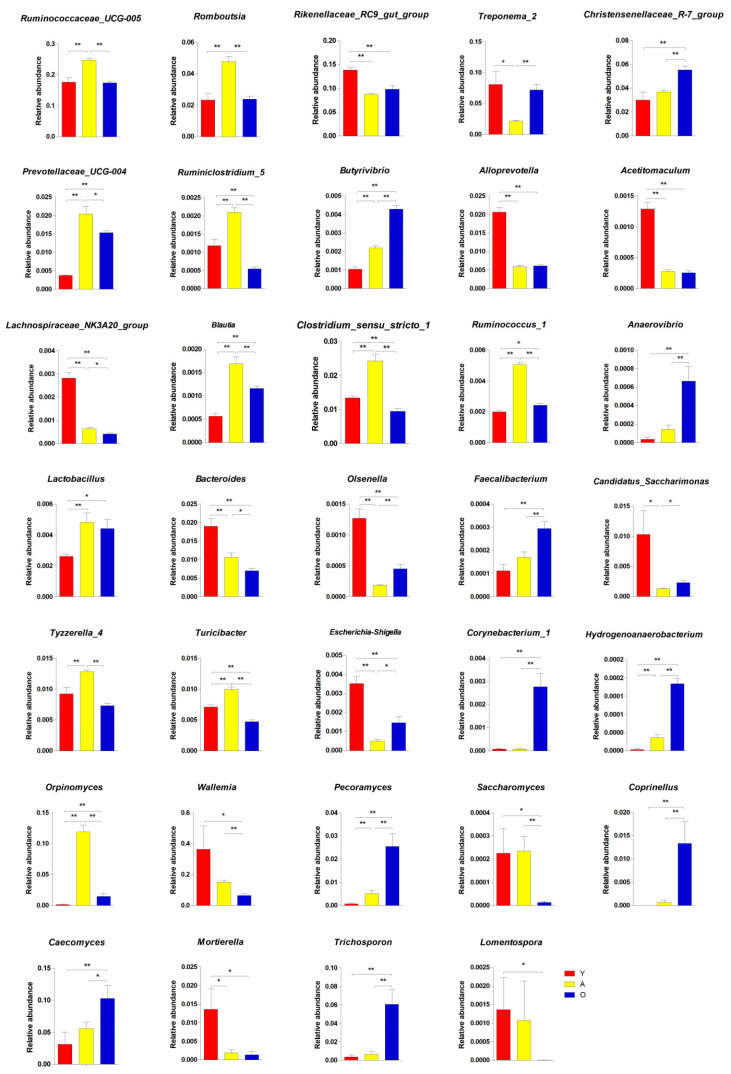
The differences in the relative abundance of bacteria and fungi between three development stages at the genus level. * *p* < 0.05; ** *p* < 0.01.

We analyzed the relative proportion of preponderant taxa of the gut bacterial community at different taxonomical levels (Figure 4A,B). Following the phylum taxonomical level assignment results, Firmicutes and Bacteroidetes were the dominant phyla regardless of age, which consisted of over 84% of total sequences. Metastats analysis was used to examine the differences in phyla among groups in order to undertake exploratory changes in all taxa as the yaks matured. To further perform an exploratory alteration in all taxa as the yaks aged, Metastats analysis was performed to compare the difference of phyla among groups (Figure 5). A continuous increase was recorded in the relative abundances of Tenericutes, Cyanobacteria, and Planctomycetes with age. Bacteroidetes, on the other hand, exhibited substantial decreases in relative abundance as the yaks matured. Similarly, enrichment of phyla Firmicutes and low abundance of phyla Proteobacteria and Spirochaetes existed in the adulthood of yaks (*p* < 0.05 or *p* < 0.01). Using genus-level cluster analysis, a total of 194 genera were identified across all data. Several abundances in the genus were among the genera found as having significant age-related variations (Figure 6). Six taxa belonging to genera *Christensenellaceae_R-7_group*, *Butyrivibrio*, *Anaerovibrio*, *Faecalibacterium*, *Corynebacterium_1*, and *Hydrogenoanaerobacterium* were significantly enriched in old age, and the majority of these also increased with age. Conversely, an abundance of *Rikenellaceae_RC9_gut_group*, *Alloprevotella*, *Acetitomaculum*, *Lachnospiraceae_NK3A20_group*, *Bacteroides*, *Olsenella*, *Candidatus_Saccharimonas*, and *Escherichia-Shigella* were negatively associated with ageing; their abundance was increased in the young yaks compared with the other two development periods. The high signals of the A group of bacteria were significant at these genera level (*Ruminococcaceae_UCG-005*, *Romboutsia*, *Prevotellaceae_UCG-004*, *Ruminiclostridium_5*, *Blautia*, *Clostridium_sensu_stricto_1*, *Ruminococcus_1*, *Lactobacillus*, and *Tyzzerella_4*, *Turicibacter*). Furthermore, *Treponema_2*, *Olsenella*, *Candidatus_Saccharimonas*, and *Escherichia-Shigella* were significantly underrepresented in adult yaks compared with young and old yaks (*p* < 0.05 or *p* < 0.01).

### 3.4. Significant Alterations in Gut Fungal-Microbiota Composition with Age

The findings of taxonomic classification of the fungal OTU preponderant taxa revealed three dominating phyla at the phylum level (Basidiomycota, Ascomycota, and Neocallimastigomycota), accounting for approximately 76% of identified taxonomic sequences (Figure 4C). When comparing all taxa among groups, we confirmed five phyla with significant changes in relative abundance, including Basidiomycota, Ascomycota, Neocallimastigomycota, Chytridiomycota, and Mortierellomycota (Figure 5). A negative association was found between the Mortierellomycota relative abundance and ageing, while the abundance of Chytridiomycota increased as yaks aged. In addition, the abundance of Neocallimastigomycota was significantly enriched in adult yaks. Meanwhile, the abundance of Ascomycota and Basidiomycota decreased first and then increased across the developmental stages of yaks. We compared relative abundance and identified several fungal genera that were significantly shifted across the developmental stages (Figure 6). *Pecoramyces*, *Coprinellus*, *Caecomyces*, and *Trichosporon* phyla were continuously enriched across all development periods, while *Saccharomyces* and *Lomentospore* were decreased significantly in old age (*p* < 0.05 or *p* < 0.01). *Wallemia* and *Mortierella* were detected to have the highest abundance in the Y group, indicating that these might have special significance at a young age (*p* < 0.05 or *p* < 0.01). Extremely significant, *Caecomyces* was enriched in adult yaks (*p* < 0.01).

### 3.5. LEfSe Analysis of Samples between Groups

**Figure 7:**
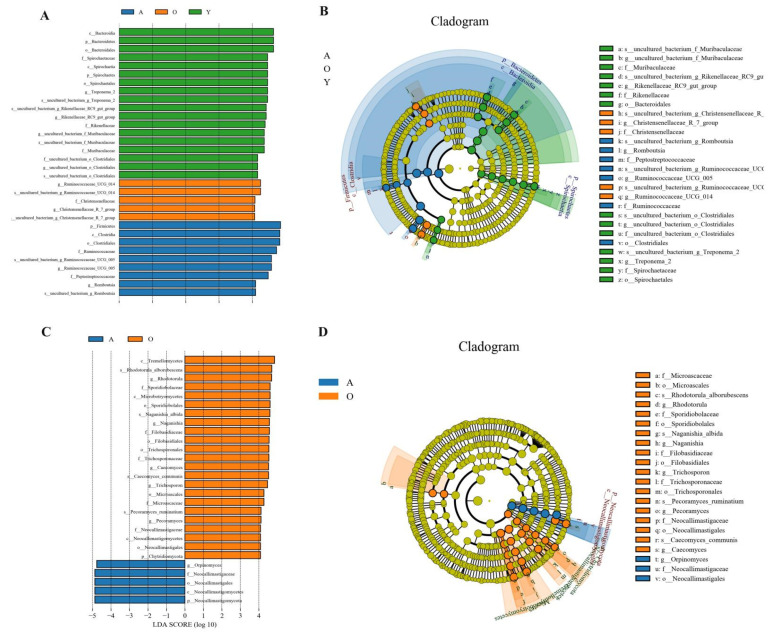
LEfSe analysis for remarking the significantly abundant bacterial and fungal microbes across development periods in yaks. (**A**,**B**) Represents bacterial analysis; (**C**,**D**) represents fungal analysis.

LEfSe was used to investigate bacterial and fungal abundance differences across development phases (Figure 7). Noteworthy changes in the microbiomes of three development periods were found (LDA Score > 4). *Treponema_2*, *Rikenellaceae_RC9_gut_group*, *uncultured_bacterium_f_Muribaculaceae*, and *uncultured_bacterium_o_Clostridiales* significantly enriched in the Y group, whereas group A (*Ruminococcaceae_UCG-005* and *Romboutsia*) and O (*Ruminococcaceae_UCG-014* and *Christensenellaceae_R-7_group*) had two bacterial genera enriched, respectively (Figure 7A,B). Six different fungal genera were collectively found between A and O, including *Rhodotorula*, *Naganishia*, *Caecomyces*, *Trichosporon*, and *Pecoramyces* in the O group *Orpinomyces* enriched in A group (Figure 7C,D).

## 4. Discussion

The interactions between the gut microbiota and the host have been identified as major modulators in disease development and progression. Studies have been carried out to see any links between changes in bacterial communities and disease, e.g., obesity, hypertension, and chronic kidney disease [32,33]. However, most microbiome research has focused on bacteria without considering the multi-kingdom nature of the microbial ecosystem. As an integral part of microbiomes, the functional ecology of fungi is not well understood. Using 16S rDNA and ITS high-throughput sequencing, we characterized the bacterial and fungal communities’ shifts in yaks across all development stages. The results revealed that the structure and compositions of the microbiome were changed dynamically in different development stages, which contribute to the maintenance of the intestinal environment’s stability, supporting the growing needs of various developmental stages, and realizing a mature functional intestinal microbiome.

Intestinal microbiome research has yielded findings and resources that link host-gut microbiome interactions to health-related consequences. Previous analysis suggested that the young animal’s diarrhea has high morbidity, and this problem eases gradually with age [34]. Moreover, this study showed that the intestinal bacterial and fungal communities significantly shift in different development stages. The alpha indices reflecting the abundance and diversity of bacterial and fungal communities significantly increased with age. As a result, we hypothesized a connection between the structure and composition of the gut microbiota that might explain why yaks of young ages are more susceptible to intestinal illness than adults. Furthermore, three branches in an evolutionary tree were sequestered with age, showed that microbiota composition and structural segregation occurred in yaks at various phases of development. This finding supported the hypothesis that each growth stage likely has a distinct selection of unique microbial communities that coexist with the host.

As previously mentioned, various beneficial microflora is positively involved in regulating gut function, host immune system and reducing incidences of intestinal diseases [35,36]. This information conveyed the message that the reduction in potential beneficial microflora abundance exacerbates intestinal disease susceptivity or that the intestinal environment, which more susceptible to disease, drives the reduction of beneficial microorganisms. This study’s results showed a continuous increase in the relative abundances of phyla Tenericutes, Cyanobacteria, and Planctomycetes with age, whereas phyla Bacteroidetes had the opposite trend. The discovery of Planctomycetes was first reported in 1924 [37]. It lives in the intestine of animals, playing a pivotal role in carbon and nitrogen cycles [38].

Similarly, to previous research, the relative abundances of Tenericutes increases as the host ages [39]. We observed that the gut microbiota of young yaks contained enriched Bacteroidetes, which is known to be involved in the production of short-chain fatty acids and is beneficial to drive the host’s intestinal immune system [40]. This assists colonization by providing a potentially advantageous way to fulfil the demands of fast growth and development. Cyanobacteria and Spirochaetes significantly enriched in old yaks, which might be a dangerous signal because bacteria belonging to these two phyla pose a big threat to animal health [34,41]. In this study, phyla Firmicutes and Proteobacteria were respectively regarded as dominant in the intestines of adult yaks. To our knowledge, phyla Firmicutes contributes to the digestion of plant fiber [42], while Proteobacteria mainly comprises Gram-negative pathogenic bacteria, including *Escherichia coli* and *Helicobacter pylori* etc. [40]. These results suggested that the microbiome gradually reaches a balanced and mature state with age.

At the genus level, the dominant microbiota in the early stage of yaks (Y group) were *Rikenellaceae_RC9_gut_group*, *Alloprevotella*, *Acetitomaculum*, *Lachnospiraceae_NK3A20_group*, *Bacteroides*, *Treponema_2*, *Olsenella*, *Candidatus_Saccharimonas*, and *Escherichia-Shigella*. Genus *Escherichia coli* was a crucial inducer of diarrhea in young ruminants, whose abundance usually increased with a high incidence of diarrhea [43,44]. However, the animals in our experiment were healthy and had no symptoms of diarrhea, indicating that the structure of the intestinal flora in the young stage might still be unstable. *Rikenellaceae* was associated with limiting inflammation in the host [45], and this process was achieved by stimulating T-cell regulatory differentiation. It was reported that *Alloprevotella* produces large amounts of succinate [46], whose number was negatively correlated with cardiovascular disease [47]. The short-chain fatty acids produced by *Acetitomaculum*, *Olsenella*, and *Lachnospiraceae* are beneficial for maintaining the function and morphology of intestinal epithelial cells [48,49,50]. Similar to the previous study, the relative abundance of *Treponema* enriches the young host’s gut, indicating that it could adapt to the young gut conditions and might be beneficial for the host [31]. As an intestinal symbiotic bacterium, *Candidatus* spp. plays an important effect in driving a mature host’s immune system [51], while a large abundance of *Bacteroides* could cause endogenous infection when the immune system is dysfunctional. These results might convey a good message that the young yak evolves towards a more mature gut community [22]. Dominance shifts of several bacteria were observed in an adult community. The percentage of *Ruminococcaceae_UCG-005*, *Romboutsia*, *Prevotellaceae_UCG-004*, *Blautia*, *Clostridium_sensu_stricto_1*, *Ruminococcus_1*, *Ruminiclostridium_5*, *Turicibacter*, and *Tyzzerella_4* in adult yaks were significantly enriched compared with the other two development stages. As mentioned by previous analysis, several members of Firmicutes (*Blautia*, *Clostridium*, *Ruminococcus*) were involved in producing SCFAs (short-chain fatty acids), which are beneficial for regulating systemic immunity [52,53]. The genome analysis of *Romboutsia* indicated the containment of genes associated with metabolic and carbohydrate utilization [54]. According to report, the abundance of *Turicibacter* increased during enteritis. However, it was not identified which disease it should be responsible for [55]. *Prevotellaceae*, a member of phylum Bacteriodetes, is mainly responsible for the digestion of plant carbohydrates, hemicellulose, pectin, etc. [56]. The presence of these microorganisms in the intestines aids adult hosts in obtaining more energy and may play a critical role in the host’s survival in the harsh natural environment. Most microorganisms were early settlers and continue to exist throughout the development period, but their abundance and proportion changed in different growth stages. The signature microbiota in the old yaks were *Christensenellaceae_R-7_group*, *Hydrogenoanaerobacterium*, *Butyrivibrio*, *Parabacteroides*, *Alistipes*, *Corynebacterium_1*, *Faecalibacterium*, *Anaerovibrio*, and *Lactobacillus*. *Christensenellaceae* is involved in the positive regulation of the gut healthy homeostasis and immunomodulation, so it has been seen as a potentially beneficial bacterium [57]. *Butyrivibrio* is commonly found in the intestines of ruminants. It digests and degrades cellulose, starch, and polysaccharides, producing a variety of short-chain fatty acids, such as acetic acid and butyric acid [58]. *Lactobacillus* and *Faecalibacterium* play a crucial role in maintaining micro-ecological balance and preventing intestinal bacterial diseases [59,60]. Both *Parabacteroides* and *Anaerovibrio* are producers of short-chain fatty acids, which maintain normal gut permeability and intestinal physiological functions [61,62]. *Hydrogenoanaerobacterium* is associated with obesity [63]. Disease indicates an imbalance between energy intake and metabolic consumption in animals. Research showed an age-related increase in skeletal muscle bioenergetics consumption during dynamic exercise [64]. *Corynebacterium* is rarely considered a pathogen. However, it is often isolated from patients with orthopedic infection [65]. A meaningful study reported that adding microorganisms from old mice (not young) to germ-free mice caused inflammation, which indicated that microorganisms gradually become harmful to the host with age [66]. Although the elderly samples we collected came from healthy yak, we found several microbial components correlated to frailty [66]. Specific bacterial metabolic function interventions were performed on bacterial strains linked to the nematode *Caenorhabditis elegans*, such as suppressing bacterial folate production and thereby prolonging the life of the animals [67]. Interestingly, a study of the gut flora of over-hundred-year-olds discovered that numerous bacteria linked to health were colonized [68]. It was hard to apply this knowledge to the microbiome of animals, but these findings showed potential strategies for manipulating the gut flora to preserve health in old age.

Fungal communities were dedicated to the decomposition of lignocellulose in ruminants, and this functionality was implemented through physical penetration and the secretion of cell wall degrading enzymes. The final products of this process were mainly acetate, formate, and hydrogen [69]. More than 400 species of fungi were identified, and they were mainly members belonging to three phyla classification levels: Chytridiomycota, Ascomycota, and Basidiomycota [70]. Neocallimastigomycota was found to have a lower abundance of diarrhea in the host than healthy animals [28]. Our study’s most abundance of them was recorded in adult yaks (*p* < 0.05 or *p* < 0.01). Our results might be related to their physiological effects in the host, such as the ability of fungi in this phylum to depolymerize complex molecular structures, degrade lignocellulosic biomass, and improve feed digestibility [71]. Previous studies only identified 18 anaerobic fungal genera from herbivores [72,73]. However, more fungal genera were identified in our research, which is likely to be linked to the following factors: First, the abundances and diversity of fungal communities are quite different between different species; secondly, yaks grow in a relatively harsh environment, which means that a more complete and mature gut microbial community is needed to cope with extreme climates (cold, hypoxia) to survive. A high abundance of *Mortierella* was identified in calves with diarrhea [28]. Species of *Lomentospora* was an emerging opportunistic pathogen, and it was shown to be a potential threat to hosts with low immune function and weakness [74]. In this study, the fungal communities significantly increased, included *Mortierella* and *Lomentospora*, in young yaks compared with the other two stages, suggesting that yaks in the early stage had an immature intestinal microbiome. *Orpinomyces* was reported for its outstanding contribution to the degradation of cellulose [75], which might reveal the up-regulation of plant digestion in adult yaks because the highest abundance of it was found in A group (*p* < 0.05 or *p* < 0.01). As a non-pathogenic species, a decline in *Saccharomyces* represents the disorder and malnutrition of the fungal flora associated with IBD [76]. The fungal genus *Wallemia* is found in various harsh environments, such as solar salterns, dry and highly sugared foods, etc. Among the eight identified species, three were related to human health problems, including *W. muriae*, *W. Sebi*, and *W. mellicola* [77]. These gradual changes in the proportion and composition of involved fungal flora (*Orpinomyces*, *Saccharomyces*, and *Wallemia*) from young to adult might be related to the more energy required by adults to maintain the steady-state of the intestinal environment under harsh environments.

Moreover, our results demonstrated a significant increase in the relative abundances of four genera (*Trichosporon*, *Coprinellus*, *Pecoramyces*, and *Caecomyces*) in the old yak’s development stage (*p* < 0.05 or *p* < 0.01). The fungal microbiome in the intestine was a powerful degrader of the plant content in the intestine of herbivorous animals [78], such as *Pecoramyces* [73]. Given that they produced a high number of biomass-degrading enzymes, these organisms have potential applications in lignocellulosic-related chemical products [79,80]. However, as an effective degrader of plant biomass, *Caecomyces* formed a limited system that could only attach to plant biomass [81]. The colonic mucosa of mice with severe colitis was often accompanied by outbreaks of opportunistic pathogenic fungi, including *Trichosporon* [76]. In this study, we found that bacteria and fungi associated with health problems increased in the intestines of aged yaks, which might confirm that the relationship between bacteria and fungi is synergistic and inter-kingdom [82].

## 5. Conclusions

In summary, our research demonstrated the dynamic distribution of gut microbiome development, including bacterial and fungal communities, in yaks during their whole development process. Specifically, the proportion and abundance of some microorganisms in young yaks tend to change and gradually merge into more mature adult components, including bacterial communities *Ruminococcaceae_UCG-005*, *Romboutsia*, *Prevotellaceae_UCG-004*, *Blautia*, *Clostridium_sensu_stricto_1*, *Ruminococcus_1*, *Ruminiclostridium_5*, *Rikenellaceae_RC9_gut_group*, *Alloprevotella*, *Acetitomaculum*, *Lachnospiraceae_NK3A20_group*, *Bacteroides*, *Treponema_2*, *Olsenella*, *Escherichia-Shigella*, *Candidatus_Saccharimonas*, and fungal communities *Mortierella*, *Lomentospora*, *Orpinomyces*, and *Saccharomyces*. Several microbial components were found correlated to frailty (*Hydrogenoanaerobacterium*, *Corynebacterium_1*, *Trichosporon*, and *Coprinellus*) in elderly samples of healthy yaks. Such colonization and succession of the host’s age-dependent intestinal microbiota may provide an important theoretical basis for microbiota-based treatment or disease prevention strategies. After all, the gut microbiota from juvenile to natural aging and the risks of various diseases based on age had a similar connection.

## Data Availability

The raw sequence data has been submitted to the NCBI Sequence Read Archive (SRA) under accession no. PRJNA745381.

## Supplementary Materials

Supplementary data is available online at https://www.mdpi.com/article/10.3390/jof7070559/s1, Figure S1: Analysis of samples feasibility in all groups, Table S1: The information of the yaks, Table S2: Statistics of samples bacterial sequenced data, Table S3: Statistics of samples fungal sequenced data.
