# Supplementary material for: Longitudinal Characterization of the Gut Bacterial and Fungal Communities in Yaks
Source: J Fungi (Basel). 2021 Jul 14;7(7):559. doi: 10.3390/jof7070559 (PMC8304987; doi:10.3390/jof7070559)
Supplement: Supplementary file 1 [file jof-07-00559-s001.zip › jof-1229502-supplementary.pdf]

Table S1. The information of the yaks.

Sample	Gender	Body weight (Kg)
Y1	Male	92.7
Y2	Male	86.1
Y3	Male	89.3
Y4	Female	78.6
Y5	Female	90.5
Y6	Female	82.8
A1	Male	424.6
A2	Male	402.4
A3	Male	443.4
A4	Female	398.7
A5	Female	401.9
A6	Female	387.6
O1	Male	294.5
O2	Male	321.9
O3	Male	301.1
O4	Female	288.4
O5	Female	310.2
O6	Female	292.2

Table S2. Statistics of samples bacterial sequenced data.

Sample ID	Raw Reads	Clean Reads	Effective Reads	Effective(%)
A1	80,074	79,163	76,252	95.23
A2	79,908	79,056	75,577	94.58
A3	80,191	79,236	75,292	93.89
A4	79,857	78,929	75,069	94
A5	71,505	70,658	67,553	94.47
A6	79,911	78,956	75,816	94.88
Y1	79,949	78,920	76,284	95.42
Y2	80,118	79,165	76,715	95.75
Y3	79,979	78,971	76,268	95.36
Y4	79,862	78,977	75,914	95.06
Y5	79,977	79,058	76,198	95.27
Y6	79,981	79,129	75,870	94.86
O1	80,022	79,073	76,491	95.59
O2	58,920	58,249	55,850	94.79
O3	78,607	77,668	75,334	95.84
O4	80,120	79,188	76,057	94.93
O5	80,261	79,420	77,644	96.74
O6	80,036	79,219	76,254	95.27

Table S3. Statistics of samples fungal sequenced data.

Sample ID	Raw Reads	Clean Reads	Effective Reads	Effective(%)
A1	73,804	71,790	71,472	96.84
A2	77,531	75,652	75,318	97.15
A3	65,032	63,258	63,111	97.05
A4	80,002	78,205	77,872	97.34
A5	66,075	64,339	63,540	96.16
A6	80,042	78,252	77,969	97.41
Y1	80,043	78,918	78,873	98.54
Y2	79,575	78,360	77,704	97.65
Y3	79,868	78,643	78,432	98.2
Y4	79,971	79,223	79,217	99.06
Y5	79,846	79,194	79,191	99.18
Y6	79,863	79,139	78,553	98.36
O1	80,169	78,725	78,249	97.61
O2	79,980	78,586	77,679	97.12
O3	80,000	78,546	77,252	96.56
O4	80,085	78,742	78,635	98.19
O5	78,435	77,178	77,033	98.21
O6	79,775	78,451	77,297	96.89

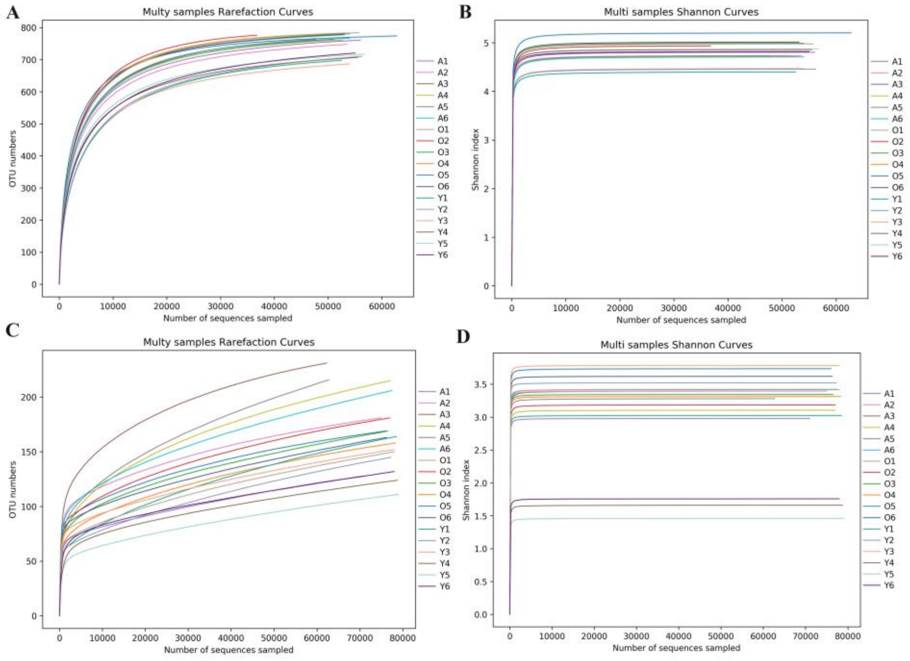

Figure S1. Analysis of samples feasibility in all groups. A, B represented Rarefaction and Shannon curves in bacterial samples, respectively. C, D represented Rarefaction and Shannon curves in fungal samples, respectively.
